# 

**Published:** 1909-02-20

**Authors:** 


### BOOKS RECEIVED.

The Caxton Publishing Co.

"The Edinburgh Stereoscopic Atlas of Obstetrics." Edited by G. F. Barbour Simpson, M.D., and Edward Burnet, B.A., M.B. Section III.

Henry Frowde and Hodder and Stoughton.

"A System of Medicine." Edited by Wm. Osler, M.D., F.R.S., and Thomas McCrae, M.D., F.R.C.P. Vol. V.

Henry J. Glaisher.

"Soured Milk and Pure Cultures of Lactic Acid Bacilli in the Treatment of Disease." By G. Herschell, M.D.

Baillière, Tindall, and Cox.

"Review of Some of the Recent Advances in Tropical Medicine, Hygiene, and Tropical Veterinary Science," by Andrew Balfour, M.D., B.Sc., F.R.C.P., Edin., D.P.H. Camb., and R. G. Archibald, M.B., R.A.M.C., attached E.A., Pathologist and Assistant Bacteriologist.

"Third Report of the Wellcome Research Laboratories," by Andrew Balfour, M.D., B.Sc., F.R.C.P. Edin., D.P.H. Camb.

Cassell and Co.

"A Manual of Medical Treatment." New edition. By J. Burney Yeo, M.D., F.R.C.P.

Longmans and Co.

"The Science of Radio Activity," by C. W. Raffety.

"The Origin of Vertebrates," by W. H. Gaskell, M.A., M.D., LL.D., F.R.S.

J Nisbet and Co.

"Injuries and Diseases of the Knee Joint," by Sir William Bennett, K.C.V.O., F.R.C.S.

"Nisbet's Medical Directory," 1909.

THE

### GRADUATES' MEDICAL SCHOOLS.

DIARY FOR THE WEEK FEB. 22 TO FEB. 27.

**THE POST-GRADUATE COLLEGE, West London Hospital, Hammersmith, W.**

**At 10 a.m.**
**Feb. 22 and 25,** Surgical Registrar, **Demonstration.**
**Feb. 26,** Medical Registrar, **Demonstration.**
**At 12 noon.**
**Feb. 22,** Dr. Low, **Pathological Demonstration.**
**At 12.15 p.m.**
**Feb. 24 and 27,** Dr. Pritchard, **Practical Medicine.**
**At 5 p.m.**
**Feb. 22,** Mr. Baldwin, **Practical Surgery—V.**
**Feb. 23,** Mr. Bidwell, **Urgent Symptoms in Abdominal Cases.**
**Feb. 24,** Mr. Pardoe, **The Prophylaxis of Venereal Diseases.**
**Feb. 25,** Mr. Lloyd, **Anæsthetics—II.**
**Feb. 26,** Dr. R. H. Cole, **Puerperal Insanity.**

**MEDICAL GRADUATES' COLLEGE AND POLYCLINIC, 22 Chenies Street, W.C.**

**At 5.15 p.m.**
**Feb. 22 and 25,** Dr. Leonard Williams, **Some Climacteric Disturbances,**
**Feb. 23,** Dr. W. S. A. Griffith, **Rupture of the Perinæum.**
**Feb. 24,** Mr. Treacher Collins, **Ocular Therapeutics.**

**NORTH-EAST LONDON POST-GRADUATE COLLEGE, Prince of Wales's General Hospital, Tottenham, N.**

**At 4.30 p.m.**
**Feb. 23,** Dr. A. H. Pirie, **The Use of X-rays in the Diagnosis of Calculi.**
**Feb. 25,** Dr. A. G. Auld, **Difficulties Encountered in the Physical Examination of the Lungs and Heart.**

**LONDON SCHOOL OF CLINICAL MEDICINE, Seamen's Hospital, Greenwich, S.E.**

**At 2.15 p.m.**
**Feb. 24,** Dr. F. Taylor, **Pheripheral Neuritis.**
**At 3.15 p.m.**
**Feb. 26,** Mr. McGavin, **Psoas Abscess: its Origin and Surgical Treatment.**

**NATIONAL HOSPITAL FOR THE PARALYSED AND EPILEPTIC, Queen Sq., Bloomsbury, W.C.**

**At 3.30 p.m.**
**Feb. 23,** Dr. Russell, **The Significance of Paralysis of Cranial Nerves.**
**Feb. 26,** Mr. Sargent, **Some Intracranial Complications of Otitis Media.**

**THE THROAT HOSPITAL, Golden Square, W.**

**At 5.30 p.m.**
**Feb. 22,** Mr. Parker, **Tuberculosis of the Pharynx and Larynx.**
**Feb. 25,** Mr. Rose, **Syphilis of the Pharynx and Larynx.**

**ST. JOHN'S HOSPITAL FOR DISEASES OF THE SKIN, Leicester Square, W.C.**

**Feb. 25,** Chesterfield Lectures, **Baldness; its Causes and Treatment.**

**LONDON THROAT HOSPITAL, Great Portland Street, W.**

**At 5 p.m.**
**Feb. 24,** Mr. Atwood Thorne, **Demonstration.**

### LONDON GENERAL HOSPITAL.

Of the four General Hospitals proposed for the London Territorial Force the first, commanded by Lieutenant-Colonel H. M. Ramsay, F.R.C.S., is to be mustered this year for its hospital training at the Cambridge Hospital, Aldershot, and the second, under Surgeon-Colonel C. Godson, at the Herbert Hospital, Woolwich. No arrangement has been made for training the third and fourth London Hospitals, which, unlike the first and second, have not yet attained the position of being officially recognised as units of the Territorial Force. The first London Hospital has twenty medical officers available for service on mobilisation and the second thirty-two, and the unrecognised third and fourth have between them sixty-four. The first London Sanitary Company, under Major L. T. F. Bryett, M.D., is to have its earliest training at the Aldershot School of Sanitation. It is understood that the trainings will be simultaneous with those of the combatant London troops in August.

### LORD GEORGE HAMILTON ON MEDICAL RELIEF.

At the annual meeting of the Ealing Cottage Hospital Lord George Hamilton, the President, expressed the hope that the Royal Commission, over which he had been presiding, to inquire into the existing administration of the Poor Law and the treatment of distress and unemployment would shortly issue its report. According to the *Times* account of the proceedings Lord George Hamilton said that, without divulging any confidence, he thought he might express his opinion upon the medical treatment of the poor, which was one of the principal things which came before them. He thought it was quite clear that more co-operation was needed between public authorities who provided medical treatment and the great voluntary organisations and hospitals which did much the same kind of work. There was a great deal of overlapping and not sufficient co-operation. His own idea was that they should try and develop contributory provident dispensaries. If they could come to an arrangement with the medical profession as to the fee they would take—that fee not being in excess of what a working man in regular employment might meet by weekly contributions—he thought they would have the foundation of a staple and satisfactory system. If they established a system on a very large scale of provident self-supporting dispensaries rather than hospitals and infirmaries where a certain proportion of the treatment must of necessity be gratuitous, and if the wage-earning class paid out of their earnings a reasonable proportion for the ordinary risk of sickness and ailment, then if any exceptional ailment or accident overcame them they could be treated to a large extent gratuitously in a hospital such as that which had been established at Ealing. If he were correct in laying down those as the fundamental conditions of relief he could congratulate the committee that at Ealing they were already working upon those lines, and that they were about to embark upon a new venture, associated with the development of which was, right from the foundation, a satisfactory and universal medical treatment for the poorer class.

### Subscriptions.

The rate of annual subscriptions to The Hospital, either direct from the Office or through agents, is :—

For the United Kingdom ... ... ... 15s. a year
To the Colonies and Abroad ... ... 17s. a year inclusive of postage in each case.

THE HOSPITAL February 20, 1909.

*Name* ..............................................................................

*Address* ..............................................................................

..............................................................................

This Coupon must accompany manuscript or contributions intended for The Hospital.

